# Dissecting the Immune Landscape of Acute Myeloid Leukemia

**DOI:** 10.3390/biomedicines6040110

**Published:** 2018-11-25

**Authors:** Jan Davidson-Moncada, Elena Viboch, Sarah E. Church, Sarah E. Warren, Sergio Rutella

**Affiliations:** 1 MacroGenics Inc., Rockville, MD 20850, USA; davidsonj@macrogenics.com; 2 NanoString Technologies Inc., Seattle, WA 98109, USA; eviboch@nanostring.com (E.V.); schurch@nanostring.com (S.E.C.); swarren@nanostring.com (S.E.W.); 3 John van Geest Cancer Research Center, School of Science and Technology, Nottingham Trent University, Nottingham NG11 8NS, UK

**Keywords:** acute myeloid leukemia, tumor immunological microenvironment, immunotherapy, immune checkpoint blockade, bispecific antibodies

## Abstract

Acute myeloid leukemia (AML) is a molecularly heterogeneous hematological malignancy with variable response to treatment. Recurring cytogenetic abnormalities and molecular lesions identify AML patient subgroups with different survival probabilities; however, 50–70% of AML cases harbor either normal or risk-indeterminate karyotypes. The discovery of better biomarkers of clinical success and failure is therefore necessary to inform tailored therapeutic decisions. Harnessing the immune system against cancer with programmed death-1 (PD-1)-directed immune checkpoint blockade (ICB) and other immunotherapy agents is an effective therapeutic option for several advanced malignancies. However, durable responses have been observed in only a minority of patients, highlighting the need to gain insights into the molecular features that predict response and to also develop more effective and rational combination therapies that address mechanisms of immune evasion and resistance. We will review the state of knowledge of the immune landscape of AML and identify the broad opportunity to further explore this incompletely characterized space. Multiplexed, spatially-resolved immunohistochemistry, flow cytometry/mass cytometry, proteomic and transcriptomic approaches are advancing our understanding of the complexity of AML-immune interactions and are expected to support the design and expedite the delivery of personalized immunotherapy clinical trials.

## 1. Introduction

Acute myeloid leukemia (AML) is characterized by bone marrow (BM) and tissue infiltration with proliferative, clonal, abnormally differentiated cells of hematopoietic origin [1,2,3,4,5]. Prognosis is currently determined by increasing age, white blood cell counts at diagnosis, cytogenetic abnormalities, and AML-specific molecular lesions. In particular, the broadly adopted 2017 ELN genetic risk stratification classifies patients with AML as being at low, intermediate, or high risk [6]. Recently, 5234 driver mutations across 76 genes or genomic regions were identified, with two or more drivers being detected in 86% of the patients [4]. Patterns of co-mutation compartmentalized the cohort of 1540 AML patients into 11 classes, each showing distinct diagnostic features and clinical outcomes. The Cancer Genome Atlas (TCGA) consortium has analyzed the genomes of 200 clinically annotated adult cases of de novo AML, using either whole-genome sequencing or whole-exome sequencing, along with RNA and micro-RNA sequencing and DNA methylation analysis [2]. Although AML genomes were shown to harbor fewer mutations than most other adult cancers, nearly all AML samples had at least 1 nonsynonymous mutation in one of nine categories of genes that are potentially relevant for pathogenesis, including transcription-factor fusions, nucleophosmin (*NPM1*), tumor suppressor genes, DNA methylation-related genes, and chromatin-modifying genes, suggesting that a complex interplay of genetic events contributes to AML development in individual patients. Importantly, a re-analysis of genetic data from 1540 patients with AML has shown that one-third of the patients may have survival predictions that deviate more than 20% from their ELN risk category [7]. Considerable efforts are thus underway to refine the accuracy of stratification algorithms and to integrate genomics findings into transformative therapeutic approaches, especially for patients with high-risk and/or refractory disease [8,9]. For example, gene expression signatures capturing leukemia stem cell-related biological pathways have been reported to identify AML patients at risk of relapse [10].

Large, systematic big data efforts that make use of publicly available resources have dramatically advanced our understanding of cancer biology and the immune landscape of solid tumors, and, by identifying genes with a key role in patient survival, are paving the way to cancer precision medicine. A systems-level, knowledge-based approach has enabled the analysis of the genome-wide transcriptome of the protein-coding genes of 17 major cancer types with large numbers of patients available in TCGA accompanied by clinical metadata (www.proteinatlas.org/pathology) [11]. Gene expression varied considerably in individual tumors within a particular cancer subtype. Also, there was a large overlap in gene expression among individuals with different cancer types. Shorter patient survival was associated with up-regulation of genes involved in cell growth and with down-regulation of genes mediating cellular differentiation. A detailed analysis revealed that no prognostic genes were shared among more than seven of the solid tumor types. Furthermore, cancer patients showed widespread metabolic heterogeneity. The observation that no general prognostic gene necessary for clinical outcome was applicable to all cancers [11] further highlights the need to advance our understanding of the specific genetic landscape of AML, with a view to inform personalised treatment decisions. In silico approaches have also been used to dissect the immunogenomic features of 33 cancer types and their therapeutic and prognostic implications, leading to the identification of six immune subtypes, i.e., wound healing, IFN-γ-dominant, inflammatory, lymphocyte depleted, immunologically quiet, and TGF-β-dominant, characterized by differences in macrophage or lymphocyte signatures, T helper type 1 (Th1):Th2 cell ratio, extent of intratumoral heterogeneity and neoantigen load, aneuploidy, cell proliferation, expression of immunomodulatory genes, and patient survival [12]. Another in silico approach relies on data from TCGA to examine immune phenomenon associated with cytolic activity using gene expression measured by RNA-seq, as shown in analysis of 18 solid tumor types [13]. The authors used two tightly co-expressed genes to identify cytolitic immune effector activity, granzyme-A and perforin, and defined differences in cytolytic activities across tumor types. The highest levels of cytolytic activity were detected in kidney clear cell carcinoma and cervical cancers. Consistent with the hypothesis that an initial immune response to solid tumors is subsequently suppressed, cytolytic activities and expression of IFN-stimulated chemokines (CXCL9, CXCL10 and CXCL11) were associated with markers of immune resistance or an immuno-suppressive TME, including IDO1, IDO2, PD-L2, and the C1Q complex. Increased cytolytic immune activity was associated with a small survival benefit across solid tumor types examined in TCGA. The lack of a more significant survival benefit may be associated with the compensatory immuno-suppressive response [13].

Cancer immunotherapy is revolutionizing the approach to human malignancies by showing that immune cells can be harnessed to destroy cancer cells in a proportion of patients [14]. Adoptive cell immunotherapy has been defined “Advance of the Year 2018” by the American Society of Clinical Oncology [15]. A deeper understanding of baseline immunity, both in the periphery and in the tumor microenvironment (TME), and of immune escape mechanisms is driving the identification of biomarkers that are predictive of clinical outcomes. These approaches are expected to also elucidate why cancer patients might fail to respond to immunotherapy [16,17]. Evidence from clinical trials indicates that pre-existing immunological features contribute to the ability of patients with solid tumors to respond to immune checkpoint blockade (ICB) [18]. Three immune profiles have been revealed by clinical studies in melanoma [19,20]. The immune-inflamed phenotype is characterized by the presence of both CD4^+^ and CD8^+^ T cells, often accompanied by myeloid and monocytic cells, and by staining for Programmed Death Ligand (PDL)-1 on tumor-infiltrating lymphocytes (TILs) and, in some cases, on tumor cells [21]. In general, patients with ‘inflamed’ tumor lesions are more likely to respond to ICB, suggesting that immunotherapies are effective by potentiating ongoing immunity rather than generating de novo immunity against cancer [22]. In tumors with an immune-excluded phenotype, which are unlikely to respond to immunotherapy, the adaptive immune cells do not reach or infiltrate the tumor. Instead, the immune system is locally suppressed, the tumor is tolerant and immune cells remains in the surrounding stroma. The third immune-desert phenotype lacks evidence of an initial immune response or subsequent creation of an immunosuppressive microenvironment. The absence of CD8^+^ T cells, myeloid-derived suppressor cells, M2 macrophages and regulatory T cells characterized this profile. The immune-desert phenotype and the immune-excluded phenotype can both be considered as non-inflamed tumors [19]. The importance of pre-existing, clonally restricted CD8 T-cell responses and of physical proximity between PD-1^+^ and PD-L1^+^ cells in the TME for tumor regression after immunotherapy with PD-1 blocking agents has again been demonstrated in patients with metastatic melanoma [23].

One important mechanism of resistance to ICB in solid tumors is the up-regulation of PD-L1, which can cause T-cell exhaustion. However, PD-L1-independent mechanisms of resistance to ICB exist, as suggested by the observation that up to 50% of PD-L1-expressing tumors are either resistant or relapse after PD-1/PD-L1 blockade [24]. Insensitivity to T-cell effector molecules, including IFN-γ, is increasingly recognized as a mediator of immunotherapeutic resistance [24]. Loss of IFN-γ signaling through inactivating mutations of JAK1 and JAK2 promotes resistance to PD-1 blockade in melanoma and mismatch repair-deficient colon cancer [25,26]. Intriguingly, prolonged treatment of B16 melanoma cells with IFN-γ has been shown to confer resistance to radiotherapy and anti-CTLA-4 immunotherapy as a result of STAT1-related epigenetic events leading to up-regulation of immunosuppressive effectors and T-cell inhibitory receptors and their ligands [27]. After reviewing the structure and functional features of the TME of patients with AML, this article will focus on recently identified targets for immune intervention and will highlight how this knowledge could be used for rational decision-making and for delivering personalized immunotherapies.

## 2. The Tumor Immunological Microenvironment

The TME is a complex milieu that is increasingly recognized as an essential determinant of tumor progression and responses to therapy, including ICB targeting the PD-1/PD-L1 inhibitory axis [28]. Tumor and stromal cells activate protein and gene expression patterns in the TME that promote tumor growth and are intrinsically immune suppressive [16,29]. Across thousands of solid tumors, RNA-seq gene expression data from TCGA indicates that a robust immune response and immune infiltration by T cells and B cells, including CD8^+^ T cells and CD45RO^+^ memory T cells as well as increased B-cell receptor (BCR) diversity are associated with an overall survival benefit. The immune gene signatures with the greatest association with survival were in the leukocyte family and macrophage signatures had a negative prognostic association. This is shown is a recent study where the authors measured the presence or absense of various immune cell populations using numerous gene expression signatures from previously published literature [30]. The geographic co-localization of PD-1/PD-L1 expression with an “inflamed” TME suggests that PD-L1 is locally up-regulated by IFN-γ in the context of an endogenous anti-tumor immune response [22]. Melanoma regression after therapeutic ICB with pembrolizumab requires pre-existing CD8^+^ T cells and phosphorylated STAT1 expression at the invasive margin [23]. A non-inflamed melanoma TME has been correlated with failed recruitment and activation of Batf3-lineage dendritic cells [31]. Immune exclusion might also be the result of tumor-intrinsic activation of specific oncogenic pathways, such as WNT/β-catenin, and PTEN deletion/PI3K activation [32,33].

PD-L1 expression in response to cytokine stimuli, most importantly IFN-γ, has been termed “adaptive or compensatory immune resistance” [34]. IFN-γ is predominantly produced by activated T cells, NK cells and NKT cells, activates downstream signaling intermediates in macrophages/DCs, including *STAT1*, and up-regulates MHC molecules and other components of the proteasome and antigen-presenting cell machineries. However, IFN-γ also mediates feedback inhibition and up-regulates PD-L1, PD-L2 and other checkpoint molecules such as IDO1, TIGIT, and LAG3, thus allowing cancer cells to survive [35]. In addition to immune effects, high levels of IFN-stimulated genes (ISGs), including *STAT1*, *IFIT1,* and *ISG15*, promote resistance to radiation and chemotherapy in a variety of solid tumors [36,37]. Particular IFN-stimulated genes in the IRDS, such as *ISG15*, have previously been involved in DNA repair [38]. This IFN-related DNA damage signature (IRDS) specifically increased the accuracy of outcome prediction in breast cancer patients receiving adjuvant chemotherapy but was not a prognostic marker [36]. Intriguingly, inflamed TMEs as defined by a 13-gene T-cell signature did not correlate with the number of mutational neoepitopes in melanoma and in other 30 solid tumor types [31]. The lack of correlation of inflamed TMEs as defined by an 18-gene Tumor Inflammation Signature with mutational burden was shown in solid and hematologic malignancies [39]. This analysis also highlighted the distinct behavior of certain immune genes in cancers arising from “immune” cells included in the TCGA database, i.e., thymoma, AML and diffuse large B-cell lymphoma (DLBCL). CD276, IDO1, and NKG7 in AML diverge from the pan-cancer trends in intercept and slope of expression observed in non “immune” derived tumor types, highlighting the need for AML-specific understanding of the TME.

The TME represents an emerging consensus view as an area with the potential for identifying novel biomarkers [40,41]. In a study of colorectal carcinoma (CRC), tumors that lost IL-15 expression had lower levels of immune activation as measured by T and B cell abudance. This immune-desert like profile was associated with worse outcomes in this patient population [42]. Further work by this group characterized immune cell types using mRNA transcripts to categorize the TME in CRC. They found the immune landscape to be diverse and to vary based on disease stage, with the most active immune response in earlier stage disease (T1) and the lowest level of immune response in advanced disease (T4). The immune infiltrate in CRC has been observed to include both innate and adaptive immune cells measured by gene expression [43]. As demonstrated in multiple prior studies of the majority of solid tumors, the immune landscape is prognostic with an adaptive immune response associated with a favorable prognosis as indicated by presence of T cells, including genes encoding molecules expressed on γδ T cells, cytotoxic T cells and follicular helper T cells, as well as macrophages, mast cell, and B cells. Immune tolerance is associated with a negative prognosis in CRC, particularly cell types such as eosinophils, Th2 cells, Th17 cells, regulatory T cells (Tregs) and natural killer (NK) cells.

A growing body of clinical observations indicates that, in addition to increased tumor immunogenicity as a result of higher mutational burden, single tumor-suppressor genes can be associated with the immune landscape of the tumor [44]. The first tumor-intrinsic oncogene pathway that can abrogate the induction of T-cell responses, prevent the establishment of a T-cell-inflamed TME and generate resistance to ICB has been described in a mouse model of melanoma [32]. Tumors expressing β-catenin showed lack of recruitment of the Batf3-lineage DCs expressing the surface markers CD103 or CD8α as a result of insufficient production of the critical chemokine CCL4 by the melanoma cells. Importantly, 50% of human non-T-cell-inflamed melanoma lesions showed evidence for activation of WNT-β-catenin signaling specifically in the tumor cells [32,45]. Links between distinct abnormalities in tumor drivers and composition of the immune cells in the TME have recently been identified in patients with prostate cancer using in silico approaches [46]. PMN and monocyte signatures derived from published papers clustered the TCGA samples into three groups with high, mid and low gene expression levels. Additional analyses of a public dataset of metastatic prostate cancer cases revealed that only 10% of patients with low PMN infiltrate harbor altered *PTEN* and *ZBTB7A* expression. Similarly, only 10% and 5% of patients with *PML* deletions clustered in the PMN-high and T-cell-high subtype, respectively. Mutations in the genes encoding isocitrate dehydrogenase 1 and 2 (*IDH1* and *IDH2*) are associated with reduced T-cell infiltration in lower-grade gliomas through diminished STAT1 signaling and lowered the expression of CXCL10, an IFN-γ-inducible chemokine mediating T-cell recruitment [47]. In 2017, enasidenib, an *IDH2* kinase inhibitor, was approved by the US Food and Drug Administration (FDA) for patients with relapsed/refractory mutant *IDH2* AML [48,49]. The impact of oncogenic pathways on the immune landscape of AML has not been explored yet. It would be important to assess whether the quality of T-cell infiltration in the AML TME is affected by the *IDH2* inhibitors currently in clinical development.

## 3. High-Resolution Platforms to Decipher the Complexity of the TME

Innate and adaptive immune responses within the TME are increasingly being assessed by gene expression profiling [50]. Immune gene signatures, especially those induced by IFN-γ, may be powerful biomarkers of response to ICB. For instance, a 10-gene IFN-γ score, including genes encoding IDO1, LAG3, PRF1, GZM and other immune-related genes, has been shown to correlate with best overall response (OR) and PFS in patients with advanced melanoma and with other solid tumors, and to be non-significantly associated with OS [51]. The Tumor Inflammation Signature (TIS) is an 28 gene assay (18 targets + 10 housekeepers) that measures the presence of an activated but suppressed immune response in the TME by integrating transcriptional evidence of innate and adaptive immune cells, IFN signaling, and T cell exhaustion [52]. The TIS was originally developed to enrich for response to pembrolizumab in a variety of solid tumors, has since been deployed in a number of retrospective studies and found to be predictive for other immunotherapies checkpoints (nivolumab, ipilimumab plus IFN, nivolumab plus ipilimumab) [53,54,55]. The TIS has been analytically validated and developed for investigation use only (IUO) deployment in clinical trials.

Immune gene co-expression patterns have been used to identify a subset of high-confidence markers in 9986 solid tumor samples from TCGA [56]. Immune cell scores calculated by gene expression were compared to standard immune cell measuring assays (flow cytometry and IHC). Sixty genes were used to develop immune cell abundance scores for 14 immune cell types, where gene expression scores were highly correlated with immune phenotypes measured by flow cytometry and IHC assays in TMEs across different tumor types. A verification cohort consisting of samples collected from patients receiving anti-CTLA-4 ICB confirmed the ability of immune cell typing gene signatures to recapitulate phenotypes found by flow cytometry, emphasizing gene expression as a technique to delineate cell types [56]. Additional verification of gene expression based immune cell typing was observed in a longitudinal study comparing metastatic melanoma samples at multiple time points from patients treated with anti-CTLA-4 followed by anti-PD-1 after progression on anti-CTLA4, using a 12-marker immunohistochemistry panel and targeted gene expression profiling on the nCounter^®^ platform [57]. Interestingly, during early treatment, adaptive immune gene signatures in biopsies displayed up-regulation of cytolytic genes, HLA molecules, IFN-γ pathway effector genes, and chemokines, and were highly predictive of response to ICB. Importantly, unique gene expression profiles observed in the TME of patients receiving monotherapy with anti-CTLA-4 or anti-PD-1 antibodies provided insights into the mechanisms of action ICBs targeted to different pathways, as well as a compelling rational for designing combination immunotherapies.

Going beyond whole sample gene expression measurement of immune biology by interrogating spatial distribution of immune, tumor and stromal cells in the TME provides a complementary understating of how immune cell localization can be used as a biomarker. Spatial data can provide a more complete picture of the behavior of immune cells to promote or reduce tumor growth, and therefore help define mechanisms of excluded and desert TMEs [50]. Multiplexed immunofluorescence allows the detection of up to 8 proteins in regions of interest within the TME. Multiple fluorophores can be applied on a single tissue section and are interrogated using a multi-spectral microscope [41,58]. This technology enables a comprehensive characterization of the topography and spatial relationship between tumor cells and stromally located cells, including immune cells. Of relevance, the density of CD8^+^ T cell infiltrates in the invasive margins of melanoma lesions has been associated with expression of the PD-1/PD-L1 immune inhibitory axis and with clinical responses to anti-PD-1 immunotherapy [23]. Quantitative image analysis could also be valuable in dissecting the spatial distribution of DCs at different maturation stages within the tumor-draining lymph nodes, thus providing insights into actionable circuits of immune dysfunction [59]. NanoString Technologies Inc. (Seattle, WA, USA) has recently developed a nondestructive multiplexed immune profiling approach to measure the expression of up to 800 targets via antibodies and in situ RNA on a single FFPE tissue slide [60]. The GeoMx™ Digital Spatial Profiling (DSP) platform allows the analysis of tumor geography and quantitation of potential biomarkers from regions of interest (ROIs) identified from tissue morphology [61]. The platform utilizes UV photocleavable oligos conjugated to a cocktail of primary antibodies to stain FFPE tissue, and then ROIs are identified with fluorescent antibodies. UV The tissue within the ROI is then exposed to UV light in precise patterns determined by masking based on the fluorescent channels, and released oligos are captured and registered to a specific location on the tissue before being enumerated by standard NanoString nCounter hybridization technology. This enables digital quantitation of up to 800 proteins from a single tissue section. By exchanging the primary antibodies for oligo labeled RNA binding probes, the same process can be used to spatially resolve RNA targets as well. We used DSP to characterize the expression patterns of immuno-oncology proteins in BM biopsies from patients with newly diagnosed AML [62]. Tissue architecture was established with fluorescent antibodies to CD3 (to label T cells), CD123 (to label AML blasts), and a DNA dye. ROIs were then selected and profiled from T cell enriched and AML blast enriched areas of the tissue. T-cell infiltration was highly heterogeneous in AML patients and across distinct ROIs from individual BM biopsies. Spatially-resolved profiling identified co-expression patterns of immuno-oncology proteins in BM biopsies, including the co-localization of PD-L1- and CD8-expressing cell types. As the DSP platform is launched commercially and becomes more widely available, it is anticipated that the technologies will be extensively applied to the analysis of tumor geography and location-specific biomarker expression.

Cytometry by time of flight (CyTOF) coupled to multiplex major histocompatibility complex (MHC)-tetramer staining has been used to identify the antigen specificity of tumor-infiltrating lymphocytes (TILs) in patients with colorectal and lung cancer [63]. TILs are not only specific for tumor-associated antigens but also recognize a variety of epitopes unrelated to cancer, such as those from Epstein-Barr virus, human cytomegalovirus, or influenza virus. The bystander CD8^+^ TILs display overlapping phenotypic features with tumor-specific T cells, including the expression of the inhibitory receptors TIGIT and PD-1, but lack expression of CD39, a transmembrane extracellular ATPase catalyzing the conversion of ATP to adenosine [63]. In contrast, CD39 expression identified tumor-specific CD8^+^ TILs with transcriptomic hallmarks of exhausted T cells that have undergone tumor antigen-driven clonal expansion. Intriguingly, colorectal tumors with higher frequencies of CD39^+^CD8^+^ TILs showed gene expression profiles consistent with a T-cell-inflamed TME, including the expression of pathways associated with antigen processing and presentation. Moreover, the frequency of CD39^+^CD8^+^ TILs was higher in epidermal growth factor receptor (EGFR)-mutated lung cancers, a subgroup of patients showing low CD8^+^ T-cell densities and relatively poor responses to ICB. Overall, this elegant study exemplifies how the use of high-dimensional technologies can advance our understanding of the complex tumor-immune interactions and support the identification of novel biomarkers [64,65].

## 4. Composition of the AML TME

The BM is a primary lymphoid organ and a distinctive immunologic microenvironment that provides support for hematopoietic stem cells (HSCs) and contains most developing and mature immune cell types [66]. Landmark populations of BM-resident immune cells have been recently identified and described in mice [67]. The integration of mass cytometry data from healthy donors into this reference map revealed a similar overlay pattern between the two species [67]. In mice, clusters of DCs co-localize with naïve B cells and T cells. Conditional deletion of macrophage migration-inhibitory factor (MIF)-producing DCs translated into a profound reduction in recirculating mature B cells in the TME, but left B-cell development unaffected [68]. Plasma cells can be detected in close proximity to CXC chemokine ligand 12 (CXCL12)-abundant reticular (CAR) cells, a population of stromal cells in contact with multipotent hematopoietic progenitors [69]. More than 80% of the surviving memory CD4^+^ T cells translocate to the BM within 3–8 weeks after initiation of the immune response [70]. Memory CD4^+^ T cells are maintained in a quiescent state by stromal cell-derived IL-7, but quickly respond to activating signals and provide efficient help to B cells for affinity maturation of antibodies. In human donors, polyfunctional CD4^+^ T cells with specificity for viral pathogens encountered in childhood, or emerging after vaccination, are maintained exclusively in the BM [71]. The BM is also a niche for the most actively dividing pool of memory CD8^+^ T cells [72]. Central-memory T cells constitute the largest endogenous subset of CD8^+^ T cells in murine BM and are also prominent in human BM [73,74]. After intravenous antigen injection, naïve T cells and central-memory T cells are more efficiently recruited to the BM than effector T cells. Adoptively transferred CD8^+^ T cells specific for melanoma antigens accumulate in tumor-draining lymph nodes at early time points after vaccination with peptide-pulsed DCs and IL-2 immunotherapy, and subsequently in the BM and primary tumor site [75]. This study suggests that the BM could replenish the supply of tumor antigen-specific CD8^+^ T cells homing to primary tumors and/or primary and secondary lymphoid organs.

**Figure 1:**
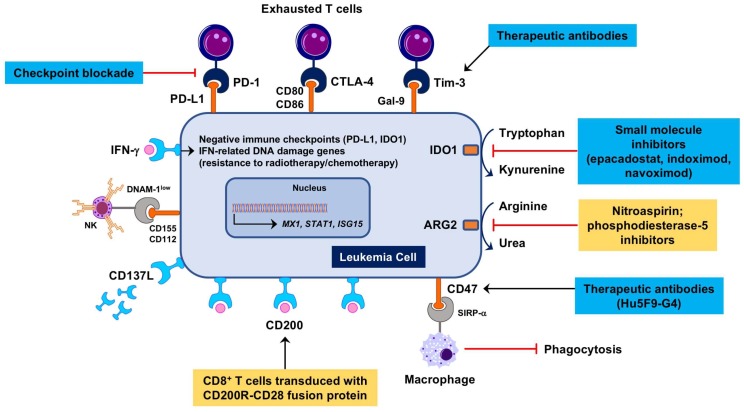
Therapeutic targeting of immune suppression in the Acute myeloid leukemia (AML) tumor immunological microenvironment (TME). Microenvironmental soluble factors, such as interferon (IFN)-γ produced by cytotoxic T cells, promote leukemia cell proliferation [85], instigate immune suppressive mechanisms, including the induction of indoleamine 2,3-dioxygenase-1 (IDO1), and mediate resistance to genotoxic damage [36]. IDO1 inhibitors such as epacadostat, indoximod and navoximod have entered the clinical arena for patients with advanced solid tumors [86,87]. Nitric oxide (NO)-releasing aspirin (nitroaspirin) interferes with the inhibitory enzymatic activities of arginase-2 (ARG2) and NO synthase expressed in myeloid cells and has been administered orally to normalize the immune status of tumor-bearing mice [88]. In AML patients, DNAM-1, an activating receptor for NK cells and T cells, is reduced and its ligands CD155 and CD112 are increased, indicating a tolerogenic phenotype [89]. Shedding of CD137L leading to increased serum levels correlates with worse prognosis and may constitute an immune suppressive circuit in AML [90]. CD200 (OX2) is a negative regulator of T-cell function that is frequently increased in AML and is associated with poor prognosis. CD200R immunomodulatory fusion proteins (IFPs) with the cytoplasmic tail replaced by the signaling domain of the costimulatory receptor CD28 have been recently engineered [91,92]. Adoptive therapy with CD200R-CD28-transduced leukemia-specific CD8^+^ T cells has been shown to eradicate murine AML more efficiently than wild-type T cells. Antibodies targeting CD47, an inhibitory receptor preventing phagocytosis of AML cells [93], are currently being tested in a phase I, dose-escalation clinical trial (ClinicalTrials.gov Identifier: NCT02678338). Antibodies targeting Tim-3 [94] are under evaluation in combination with hypomethylating agents and immune checkpoint blockade (ICB) for patients with AML and high-risk myelodysplastic syndrome (MDS) (ClinicalTrials.gov Identifier: NCT03066648). Blue boxes denote therapeutic strategies already in the clinic. Yellow boxes highlight therapeutic strategies that have been evaluated pre-clinically. ARG2 = arginase-2; Gal-9 = galectin-9; NK = natural killer.

In light of their origin from primary and secondary lymphoid tissues, hematological malignancies are poorly immunogenic and highly immune-suppressive [76]. AML, the main focus of this review article, constrains protective anti-tumor immune responses through multiple mechanisms (Figure 1), including the down-regulation of major histocompatibility complex (MHC) class I and class II expression, the consumption of essential amino acids through arginase-2 (ARG2) [77] and indoleamine 2,3-dioxygenase-1 (IDO1) [78], the induction of DC dysfunction, the expansion of Treg cells [79] and the up-regulation of PD-L1 and other negative checkpoint molecules, such as Cytotoxic T-Lymphocyte-associated Antigen-4 (CTLA-4) and Lymphocyte Activation Gene 3 (LAG-3) (reviewed in reference [80]). Immune responses are defective in patients with AML due to the presence of powerful immune suppressive circuits that are activated by soluble factors and immune checkpoint molecules, including PD-L1, TIM-3, and IDO1 [78,81]. Serum kynurenine and tryptophan levels at diagnosis, a measure of systemic IDO1 activity, correlate with patient outcome [82]. Importantly, genetic mutations such as t(8;21) and inv(16) directly affect the expression of CD200 (a suppressor of macrophage and NK cell function) and CD48 (the ligand for the activating NK receptor CD244), respectively. We have recently used the NanoString immune gene expression profiling platform to decipher the complexity of the AML BM microenvironment and to identify molecular determinants of AML sensitivity to IFN-γ, patient response to chemotherapy and patient survival [83,84]. We showed that AML patients with immune-enriched and IFN-γ-dominant TME, as defined by heightened expression of *CD8A*, *IFNG*, *FOXP3*, T-cell chemoattractants *CXCL9* and *CXCL10*, *IDO1* and immune checkpoints *LAG3*, *CTLA4*, and PD-*L1*, are less likely to respond to anthracycline-based cytotoxic chemotherapy, or experience significantly shorter relapse-free survival, both indicative of primary treatment refractory states [84].

Our in silico analysis of TCGA-AML cases (162 patients treated with curative intent) also showed that abnormalities in IFN-γ-responsive genes, occurring in 30% of cases and including mRNA up-regulation and gene amplification, correlated with shorter event-free survival and OS, suggesting the hypothesis that IFN-γ-dominant TME profiles, while defining resistance to cytotoxic chemotherapy, may predict response to immune based therapy [84].

## 5. Prognostic and Predictive Immune Biomarkers in the AML TME

The majority of predictive clinical biomarker studies of immune gene expression have been conducted in solid tumors, with the most substantial work occurring in areas with the largest number of approved immunotherapies and advanced clinical stage therapeutic agents in development, such as melanoma, NSCLC, and CRC. In contrast, our understanding of the immune landscape and the role of immune gene expression in prognosis and predictive biomarkers for hematological malignancies is still emerging. Hematological malignancies represent a substantial opportunity to further deliver clinical benefit, with the principal of therapeutic immune opportunities suggested by the well characterized curative benefit from allogeneic hematopoietic stem cell transplantation (HSCT). Immune checkpoint blockade (ICB), most notably anti-PD-1 and anti-PD-L1 antibodies, have recently been studies as therapeutic strategies for patients with Hodgkin and non-Hodgkin lymphoma [95,96], with nivolumab being granted accelerated approval by the US FDA on 17 May 2016 for classical Hodgkin lymphoma, and pembrolizumab being granted accelerated approval on 13 June 2018 for primary mediastinal large B-cell lymphoma. There remains a substantial opportunity to improve our understanding of patient benefit from ICB in order to exclude patients unlikely to benefit from ICB (negative predictive value) and enroll patients likely to benefit from ICB (positive predictive value). ICB may play an important role in the treatment of selected patients with leukemia, lymphoma and multiple myeloma, as well as offer a potential for combination therapy for patients who are unlikely to benefit from a monotherapy ICB approach. While numerous studies have identified promising associations with immune gene expression and prognosis, as well as response to immunotheries, there are also substantial challenges to identifying biomarkers that are clinically relevant and robust [97]. There have been some promising studies of peripheral biomarkers at baseline that are associated with progression-free survival (PFS) in the context of therapeutic vaccines for metastatic breast cancer and prostate cancer [98]. However, this work has not been correlated with tumor site biopsies or generalized beyond these specific therapeutic contexts. In addition, there are challenges with measuring peripheral immune gene expression due to a lack of strong evidence linking the peripheral immune landscape to the immune infiltrate in solid and hematological tumor sites including lymph nodes and bone marrow [41,99].

**Figure 2:**
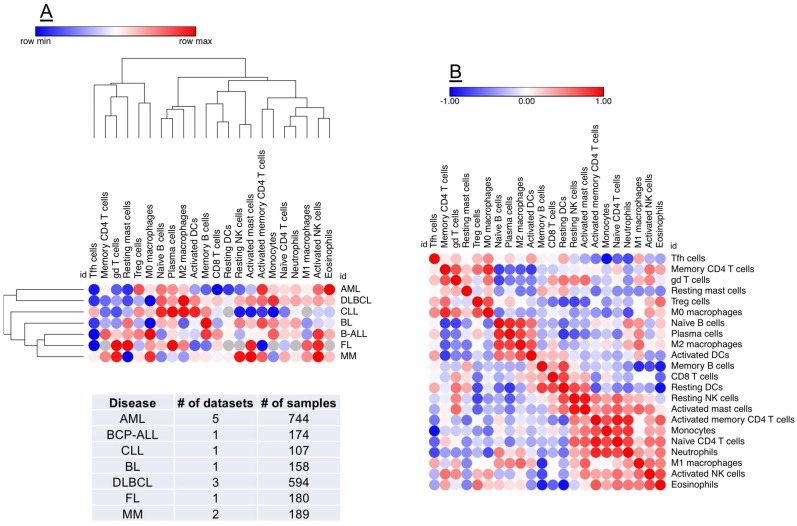
Identification of prognostic biomarkers in the AML TME. PREdiction of Clinical Outcomes from Genomic profiles (PRECOG) is a pan-cancer resource supporting the identification of prognostic genes in public datasets of human malignancies [104]. A machine-learning tool, known as CIBERSORT [102], can be applied to PRECOG data to comprehensively map compositional differences in tumor-infiltrating leukocytes (22 distinct subsets) in relation to patient outcome. (**Panel A**) shows hierarchical clustering (Euclidean distance; complete linkage) of CIBERSORT-inferred immune cell type fractions in a broad spectrum of hematological malignancies (1957 samples), including AML. Data were analyzed using Morpheus (Broad Institute, MA; https://software.broadinstitute.org/morpheus/). Red denotes an association with shorter survival times, whereas blue indicates an association with better clinical outcomes. Each column represents an immune cell type and each row represents a disease type. (**Panel B**) shows a similarity matrix (Pearson correlation) of CIBERSORT-inferred immune cell type fractions in hematological malignancies. This unbiased approach could support the identification of co-expression patterns of specific immune cell populations in the TME, thus providing unique insights into the immuno-biology of hematological malignancies and accelerating the delivery of personalized immunotherapy approaches. BCP-ALL = B-cell precursor acute lymphoblastic leukemia; CLL = chronic lymphocytic leukemia; BL = Burkitt lymphoma; DLBCL = diffuse large B-cell lymphoma; FL = follicular lymphoma; MM = multiple myeloma.

The availability of on-line tools and public collections of transcriptomic datasets has expanded our predictive capabilities [100] and could accelerate the in-silico identification of immune gene signatures and molecular drivers implicated in the progression of AML and in therapeutic responses to chemotherapy and immunotherapy [101,102,103]. Prediction of Clinical Outcomes from Genomic profiles (PRECOG; http://precog.stanford.edu) is a pan-cancer resource supporting the identification of prognostic genes in 18,000 human samples across 39 cancer types with survival outcomes, including hematological malignancies (Figure 2) [104]. One tool for evaluating the level of correlation with response is as *z*-score. The *z*-score can be generated for survival or other clinical outcomes as a measure of the correlation between the clinical outcome and gene expression, and is a normalized measure, expressing a count of the number of standard deviations from the mean. Z-scores are a powerful tool for meta-analysis because they can conveniently be combined and compared across studies. The PRECOG study has identified immune gene signatures associated with negative prognosis and identified potential mechanism of poor outcome, in this case cell proliferation [104]. The other large tumor cluster was enriched in immunological processes and immune-response genes and was associated with favorable survival. A new machine-learning tool, known as CIBERSORT [102], was also applied to the PRECOG data to explore associations predictive of clinical outcomes. Gene expression was used to characterize 22 cell types. The CIBERSORT method demonstrated abundant plasma cells in multiple myeloma and an abundance of B-cells in B-cell malignancies, demonstrating the utility of this approach in indentifying leukocyte subsets across various malignancy types [104]. When conducting a pan-cancer analysis, overall prognostic patterns correlated with leukocytes emerged: a relative abundance of T cells, particularly intra-tumor γδ T-cells, were associated with superior survival. Negative prognostic factors also emerged, with polymorphonuclear cell fractions having the most significant negative prognostic association. Finally, pro-inflammatory M1 macrophages were associated with a better clinical outcome than polarized M2 macrophages.

## 6. Immune Checkpoint Blockade and Novel Immunotherapies for “Inflamed” AMLs

Major successes with ICB are driving thousands of immunotherapy clinical trials worldwide [105]. PD-1 and CTLA-4 blockade is also being investigated in myeloid malignancies, especially MDS, as reviewed elsewhere [106]. Although AML has a low mutational burden and often exhibits an immune desert phenotype, the curative benefit of HSCT demonstrates the potential for immunotherapeutic strategies including enhancing T-cell activation. This approach may be particularly viable in patients who have an initial complete response following induction chemotherapy or have minimal residual disease such that ICB and potentially other immunotherapeutic strategies could overcome the AML-induced immune dysfunction. Ongoing trials with ICB for AML and MDS are summarized in Table 1. A recent multi-center phase II clinical trial has evaluated the feasibility and efficacy of high-dose cytarabine followed by pembrolizumab in 13 adult patients with relapsed/refractory AML [107]. In 10 evaluable patients (as of 1 July 2017), OR rates were 50%. Findings from small subgroups of responders (*n* = 3) versus non-responders (*n* = 3) documented an increased diversity of TCR Vβ repertoires in CD8^+^ T cells from responders. A recent case report documented disease-modifying activity of pembrolizumab in an AML patient with synchronous cutaneous melanoma [108]. It is presently unknown whether selecting tumors for PD-L1 expression is the best way to optimize PD-L1 blockade in hematological malignancies. Also, the definition of PD-L1 positivity depends on the sensitivity of the diagnostic antibody and on the chosen threshold. The determinants of response and resistance to ICB, including the composition and functional status of the BM TME, in AML remain to be elucidated.

Flotetuzumab (MacroGenics Inc., Rockville, MD, USA) is a bispecific CD3 × CD123 DART^®^ molecule binding T lymphocytes and cells expressing CD123, an antigen up-regulated in AML. Flotetuzumab therefore mediates AML blast killing and concomitantly activates and expands residual T cells. In monkeys, continuous infusion of flotetuzumab depleted circulating CD123^+^ AML cells as early as 72 h after treatment initiation [109]. Cytokine release, observed after the first flotetuzumab infusion, was reduced after subsequent administrations. No T-cell exhaustion was evident in animals after prolonged in vivo drug exposure. Flotetuzumab is currently being tested in hematological malignancies, including AML, with clinical activity in relapsed and refractory AML [9]. We previously reported that immune-enriched and IFN-γ-dominant gene expression profiles in the TME predicted resistance to standard chemotherapy in AML patients [84]. Interestingly, immune-enriched profiles were associated with heightened probability of response to flotetuzumab [110]. Moreover, selecting patients by response to previous chemotherapy failure, which are associated with increased IFN-γ-dominant scores, enriched complete response (CR) rates [111]. Interestingly, in responders to flotetuzumab, IFN-γ signaling scores were significantly higher at baseline (3.31 ± 0.32) than in non-responders (2.27 ± 0.11, *p* = 0.0005) and showed predictive ability (AUC = 0.815) [110]. Lastly, treatment with flotetuzumab enhanced immune infiltration and activation in the TME [110,112]. Particulary, treatment with flotetuzumab led to increased immune cell infiltrate and immune activation, as reflected by higher TIS (6.49 ± 0.20 versus 5.93 ± 0.12, *p* = 0.015), immunoproteasome (5.72 ± 0.07 versus 5.23 ± 0.10, *p* = 0.0002), and IFN-γ signaling (3.38 ± 0.23 versus 2.53 ± 0.14, *p* = 0.0015) [110], as well as increase in PD-L1 expression [112]. Primary AML blasts with higher levels of PD-L1 on malignant blasts were less susceptible to flotetuzumab-mediated killing in vitro and in vivo [113]. Hence, rational combination treatment between flotetuzumab and ICB may be an optimal design strategy to synergize anti-leukemic activity of these two agents. To this end, a study of flotetuzumab combined with MGA012, an anti-PD-1 antibody, is planned in patients with relapsed or refractory AML. Multispectral immunohistochemistry analysis of BM samples revealed a ~2-fold increase in CD8^+^ T cells upon treatment with flotetuzumab, along with the up-regulation of PD-1 on both CD4^+^ and CD8^+^ T cells [113]. Circulating levels of IFN-γ in flotetuzumab-treated patients correlated with PD-L1 expression on AML blasts. Intriguingly, primary blasts from AML cases with higher levels of PD-L1 were less susceptible to flotetuzumab-mediated killing in vitro. This study provides a compelling rationale for combining flotetuzumab with anti-PD-1/PD-L1 ICB in selected patients with relapsed or refractory AML.

## 7. Conclusions and Translational Outlook

As reviewed herein, research efforts are being devoted to the identification of ICB-responsive TME in patients with solid tumors [100]. Immune gene signatures are an emerging area with great promise for enhancing clinical decision making in patients with hematologic malignancies. As the field advances our knowledge of immunology, the dynamic and complex nature of patients’ immunological profiles has become increasingly of interest, with factors as diverse as tumor genetics, epigenetics, mRNA expression, micro-RNA expression, patient age, microbiome composition, pharmacological agents and environmental factors, including infections and exposure to sunlight, affecting patients immunologic profiles [19]. Compelling evidence now indicates patients with solid tumors who benefit from ICB typically have an inflamed immune status, with a pre-existing immune response and cytolytic markers with subsequent establishment of immune suppression by various molecular circuits that may be targeted with rational combinations of therapeutics to address distinct mechanism of immune silencing and immune escape (for example IDO1) [87]. Studies in melanoma and other solid tumors have clearly shown that IFN-γ-related mRNA profiles predict clinical response to pembrolizumab [52]. IFN-γ signatures could also identify AML patients with greater likelihood of responding to immunotherapies, including flotetuzumab, and could reveal novel targets for converting ICB-resistant tumors to a state of responsiveness.

High-dimensional technologies are enhancing our understanding of TME interactions and have the undisputed potential to support the prediction of therapeutic benefit from immune-based interventions. Because of inherent limitations of gene expression profiles, other approaches, such as flow cytometry, quantitative immunohistochemistry and next-generation sequencing for T cell antigen receptors or similar technologies (multiplex quantitative PCR, spectratyping and immune phenotyping) are recommended to thoroughly characterize the immunological landscape of the TME and to establish predictive models [23], as recently reviewed by the Immune Biomarkers Task Force of the Society for Immunotherapy of Cancer (SITC) [41]. Conceivably, systems biology approaches using multi-dimensional data analysis will be instrumental to generating the complete picture of the immunological contexture of hematological malignancies, to revealing potential immune biomarkers and informing the rational design of immune therapies. A combination of personalized transcriptomic and proteomic measurements are likely to be required to develop accurate immune signatures to give patients optimal clinical outcomes.

**Figure 3:**
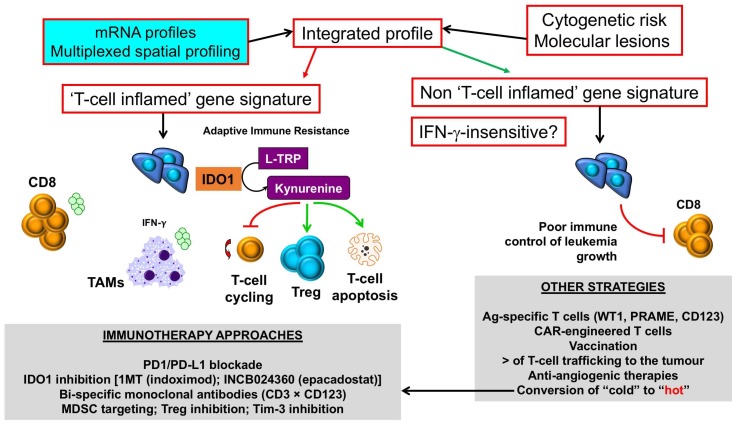
Selection of immunotherapy approaches in AML with inflamed versus non-inflamed TMEs. Messenger RNA (mRNA) profiles and spatially-resolved expression of immune checkpoints could be integrated with conventional AML prognosticators, such as patient age, presenting white blood cell count and ELN cytogenetic risk, to stratify patients into categories with different survival probabilities. Patients with T-cell inflamed profiles, indicative of adaptive resistance-driven immune dysfunction, could be considered for immunotherapy approaches that incorporate IDO1 inhibitors [86], either as monotherapy or in combination with PD-1/PD-L1 ICB [87], or other immunotherapy agents that deliver an activation signal to T cells, including CD3 × CD123 DART proteins [9], and/or revert MDSC- and Treg-mediated immune dysfunction in the TME. In contrast, AML cases with a non-T-cell inflamed TME, and/or blast cells lacking IFN-γ responsiveness as a result of abnormalities in intracellular signaling pathways, could be candidates for therapeutic strategies that enhance T-cell trafficking into the BM (STING agonists, β-catenin inhibitors [31]) and/or passive immunotherapy approaches such as the infusion of leukemia antigen-specific T cells or CD123-CAR T cells [115]. Pharmacological approaches, including the use of hypomethylating agents, could enhance T-cell infiltration to the BM, thus converting a “cold” TME into a “hot” TME [110]. IDO1 = Indoleamine 2,3-dioxygenase-1; L-TRP = l-tryptophan; 1MT = 1-methyl-tryptophan; CAR = chimeric antigen receptor; TAM = tumor-associated macrophage; Treg = regulatory T cell; MDSC = myeloid-derived suppressor cell; WT1 = Wilms’ tumor 1; PRAME = preferentially expressed antigen in melanoma. Green arrows denote stimulation; red arrows denote inhibition.

The integration of immune gene expression profiles into current algorithms for risk-stratification, which predominantly rely on ELN cytogenetic risk categories, may allow the identification of patient subgroups with “inflamed” or hot AML, who could be amenable to ICB and other immunotherapy approaches, incuding the use of bispecific CD3 × CD123 molecules and small-molecule IDO1 inhibitors (Figure 3). By contrast, AML patients with a “non-inflamed” or cold TME, or with inherent insensitivity to IFN-γ and other effector molecules, should be offered other immunotherapeutic options, such as the infusion of leukemia antigen-specific T cells and genetically-modified (chimeric antigen receptor) CAR T cells. Pharmacological strategies are also being developed to convert “cold” TMEs to “hot” TMEs. In this respect, hypomethylating agents can induce the expression of tumor-associated antigens and anti-viral cytokines, re-induce the expression of silenced endogenous retroviruses that provoke immune responses [114], and also enhance T-cell infiltration in the AML TME [110].

Integretion of multiple biomarker strateges will be imperative to capturing immune signatures within the TME that can be developed into predictive biomarkers for monotherapies and combination ICB therapeutics in hematolagic malignancies, an area that is expected to flourish during the next few years [50]. For example, gene expression profiling approaches, such as NanoString’s digital barcoding platform [60], coupled with multiplexed immunohistochemistry techniques, can be used to quantify pertinent IO genes and large numbers of proteins expressed in cell populations within localized compartments in the TME, providing crucial topography and spatial localization of immune cells at different tumor stages or after treatment with immunotherapies. High-resolution data on the composition and quality of the intra-tumor immunological infiltrate are expected to bring the next generation of immuno-oncology biomarkers to the clinic and to propel the development of novel immunotherapies [110]. For instance, a web application that has recently been developed to predict response, and intrinsic resistance, to ICB using transcriptomic profiles (http://tide.dfci.harvard.edu) may support oncologists in the selection of patients who are more likely to benefit from ICB [116].

In conclusion, recent integrative analyses of multiple human solid tumor studies comprising thousands of samples have uncovered meaningful effects of tumor-infiltrating immune cell types on patient survival and on response to ICB and other immunotherapies [117]. Future studies will evaluate whether these approaches help predict immunotherapy responses also in acute myeloid leukemia, lymphoma and other hematological malignancies, as sufficiently large patient series and datasets are accumulated and analyzed. It will also be highly relevant to assess how tumor cell-intrinsic factors, including the expression of cancer driver genes, shape the composition and quality of the TME, thus potentially influencing immunotherapy outcomes.

## Figures and Tables

**Table 1:** Completed and ongoing clinical trials of ICB with anti-PD-1/PD-L1 antibodies in AML.

Disease Stage	Therapeutic Agents	Study Design	Participants	Estimated Completion Date	Principal INVESTIGATOR	Clinicaltrials Gov. Identifier
Newly diagnosed AML age ≥ 60 years not eligible for intensive chemotherapy or HR MDS	Azacitidine monotherapy (days 1–7 every 28 days), or Azacitidine (days 1–7 every 28 days) + Nivolumab (every 2 weeks) or Azacitadine (days 1–7 every 28 days) ± Midostaurin (BID days 8–21 every 28 days), or Decitabine (days 1–5 every 28 days) and Cytarabine (days 6–11 every 28 days)	Randomized (stratified by FLT3 mutational status) Open-label Phase 2–3	*n* = 1670	August 2023	Laura Michaelis, MD	NCT03092674
Newly diagnosed AML age ≥ 60 years in first CR not eligible for HSCT	Pembrolizumab (200 mg every 3 weeks)	Non-randomized Open-label Phase 2	*n* = 40	October 2020	Michael Boyiadzis, MD, MHSc	NCT02708641
Previously untreated AML age ≥ 65 not eligible for HSCT or Previously untreated MDS	Durvalumab (1500 mg day 1 every 4 weeks) and Azacytidine (75 mg/m^2^ for 7 days every 4 weeks) vs. Azacytidine monotherapy (75 mg/m^2^ for 7 days every 4 weeks)	Randomized Open-label Phase 2	*n* = 213	April 2019	Not listed/Celgene	NCT02775903
Previously untreated AML not suitable for intensive chemotherapy	Avelumab (10 mg/kg, day 1, every 14 days) and Decitabine (20 mg/m^2^ IV days 1–5, every 28 days)	Non-randomized Open-label Phase 1	*n* = 15	December 2020	Hong Zheng, MD	NCT03395873
HR AML	Pembrolizumab on day +1 following lymphodepleting chemotherapy with FLU/MEL and autologous HSCT	Non-randomized Open-label Phase 2	*n* = 20	June 2021	Scott Solomon, MD	NCT02771197
Newly diagnosed AML age ≥ 65 years or R/R AML	Azacitidine (75 mg/m^2^ days 1–7 every 28 days) + pembrolizumab (200 mg every 3 weeks starting on day 8 of cycle 1)	Non-randomized Open-label Phase 2	*n* = 40	July 2020	Ivana Gojo, MD	NCT02845297
Newly diagnosed elderly AML (≥65 years) or R/R AML	Azacitidine + Nivolumab dose escalation starting at 75 mg/m^2^ (SQ) on days 1–7 of every 28 day cycle + 3.0 mg/kg on day 1 and day 14 every 28 days for the first 4 cycles or until CR (whichever occurs earlier) followed by a maintenance regimen (one dose of nivolumab on day 1 of each cycle of 5-azacytidine). Dose expansion with maximum tolerated dose (MTD)); or Azacitidine + Nivolumab + Ipilimumab dose escalation with Azacitidine + Nivolumab doses per above and Ipilumab starting at 1 mg/kg every 12 weeks. Dose expansion with MTD.	Non-randomized Open-label Phase II	*n* = 182	April 2020	Naval Daver, MD	NCT02397720
AML (newly diagnosed for dose-expansion; newly diagnosed or R/R for dose escalation) and HR-MDS	Idarubicin (12 mg/m^2^ days 1–3 of 28 day cycle), cytarabine (1.5 g/m^2^ days 1–4 of 28 day cycle) with Solumedrol 50 mg; or Dexamethasone 10 mg for 3–4 days on days 1–4 and nivolumab (starting dose of 1 mg/kg on day 24 of 28 day cycle and dose escalated in successive cohorts to MTD)	Non-randomized Open label Phase 1/2	*n* = 75	July 2019	Farhad Ravandi-Kashani, MD	NCT02464657
AML (newly diagnosed elderly AML unfit for induction chemotherapy and R/R for dose-expansion; R/R for dose escalation)	Atezolizumab (840 mg on days 8 and 22 of every 28-day cycle) and guadecitabine (60 mg/m^2^ on days 1–5 of every 28-day cycle)	Non-randomized Open label Phase 1b	*n* = 40	January 2019	Not listed/Hoffmann-La Roche	NCT02892318
AML (newly diagnosed AML not suitable for standard induction) or R/R AML or HR-MDS or HR-MDS who have failed hypo-methylating agent therapy	Decitabine + PDR001 (anti-PD-1) or Decitabine + MBG453 (anti-TIM3) or Decitabine + PDR001 + MBG453 or MBG453 monotherapy or MBG453 + PDR001	Non-randomized Open label Phase 1b	*n* = 175	April 2020	Andrew M. Brunner, MD	NCT03066648
AML in remission at HR for relapse	Nivolumab (3 mg/kg days 1 and 15 every 28 days, after cycle 6 day 1 every 28 days, after cycle 12 reduce to 1 time every 12 weeks)	Non-randomized Open-label Phase 2	*n* = 30	October 2020	Tapan Kadia, MD	NCT02532231
AML in remission after chemotherapy	Nivolumab (every 2 weeks for 46 courses)	Randomized Open-label, with cross-over upon relapse Phase 2	*n* = 80	June 2019	Hongtao Liu, MD, PhD	NCT02275533
Eldery AML (≥ 60 years) with CR or CRI after induction/consolidation and MRD positive status not planned for HSCT	Atezolizumab (1200 mg every cycle) and BL-8040 (1.25 mg/kg days 1–3 of cycle)	Randomized Open label Phase 1b/2 60 participants	*n* = 60	March 2022	Not listed/BioLineRx	NCT03154827
Refractory AML	Pembrolizumab (200 mg every 3 weeks)	Non-randomized Open-label Phase 0 pilot study	*n* = 10	August 2022	Michael Boyiadzis, MD, MHSc	NCT03291353
R/R AML	Decitabine (20 mg/m^2^ day 8 through 12 and 15 through 19 on alternative cycles) + pembrolizumab (200 mg; every cycle (21 days))	Non-randomized Open-label Phase 1–2	*n* = 15	July 2019	Christopher S Hourigan, MD	NCT02996474
R/R AML	HiDAC salvage induction therapy followed by pembrolizumab monotherapy on day 14 (200 mg) and every 3 weeks	Non-randomized Open-label Phase 2	*n* = 37	September 2025	Joshua F Zeidner, MD	NCT02768792
Elderly AML age ≥ 55	Cytarabine (500–1000 mg/m^2^ bid days −4, −3, −2) + G-CSF mobilized HLA-haploidentical donor peripheral blood stem cells (day 0) + Nivolumab (40 mg day +5 for 2–3 cycles) or Cytarabine (500–1000 mg/m^2^ bid days +1, +2, +3) + Nivolumab (40 mg day +1 for 2–3 cycles)	Randomized Open-label Haploidentical T cells, cytarabine and nivolumab vs. cytarabine and nivolumab Phase 2	*n* = 52	October 2020	Boris Afanasyev, MD, Prof. & Anna Smirnova, PhD	NCT03381118
AML, ALL, or MDS with relapse after allogeneic HSCT	Pembrolizumab (200 mg every 3 weeks)	Non-randomized Open-label Phase 1b	*n* = 20	October 2021	John M Magenau, MD	NCT03286114
AML and other hematological malignancies with relapse after allogeneic HSCT	Pembrolizumab (200 mg every 3 weeks for up to 24 months)	Non-randomized Open-label Phase 1 pilot study	*n* = 26	February 2020	Justin Kline, MD	NCT02981914
AML and MDS after allogeneic HSCT at HR for post-transplant recurrence	Nivolumab (1 or 3 mg/kg every 3 weeks for up to 34 weeks) or Ipilimumab (0.3 mg/kg, 1 mg/kg or 3 mg/kg every 3 weeks for up to 16 weeks) or Nivolumab + Ipilimumab (3 mg/kg every 3 weeks for up to 34 weeks and 0.3 mg/kg, 0.6 mg/kg or 1.0 mg/kg every 3 weeks for up to 16 weeks respectively)	Non-randomized Open-label Phase 1	*n* = 21	July 2023	Andrew Pecora, MD & James McCloskey, MD & Jamie Koprivnika, MD	NCT02846376
HR R/R AML following allogeneic HSCT	Nivolumab (days 1 and 15 every 28 days) up to 6 courses or Ipilimumab (day 1 every 21 days) up to 6 courses or Nivolumab (days 1 and 14 every 28 days) + Ipilumab (day 1 every 28 days) up to 6 courses	Non-randomized Open-label Phase 1	*n* = 55	January 2020	Gheath Al-Atrash, DO, PhD	NCT03600155
R/R AML and HR-MDS	Cyclophosphamide (50 mg orally) + nivolumab (3 mg/kg (or if prior alloHSCT, 1 mg/kg) every 14 days on Days 1 and 15 for up to four 28-day courses) or Cyclophosphamide (350 mg orally) + nivolumab (3 mg/kg (or if prior alloHSCT, 1 mg/kg) every 14 days on Days 1 and 15 for up to four 28-day courses)	Randomized Open-label Phase 2	*n* = 32	February 2023	Daniel J Weisdorf, MD	NCT03417154
R/R AML	PF-04518600 (anti-Ox40) monotherapy (dose escalation starting dose of 0.3 (units not given) on days 1 and 14 of a 28 day cycle) or PF-04518600 (dose escalation per above) and avelumab (10 mg/kg on days 1 and 14 of a 28 day cycle) or PF-04518600 (dose escalation per above) + Azacitidine (75 mg/m^2^ on days 1–5 or 1–7) or PF-04518600 (dose escalation per above) + Utomilumab (anti-CD137) (100 mg on days 1 and 14 of a 28 day cycle) or Avelumab (10 mg/kg on days 1 and 14 of a 28 day cycle) + Utomilumab (100 mg on days 1 and 14 of a 28 day cycle) or PF-04518600 (dose escalation per above) + Avelumab (10 mg/kg on days 1 and 14 of a 28 day cycle) + Azacitidine (75 mg/m^2^ on days 1–5 or 1–7) or Gemtuzumab Ozogamicin (3 mg/m^2^ on Days 1, 4, and 7 of each 28 day cycle) + Glasdegib (smoothened inhibitor) (100 mg oral daily) or Avelumab (10 mg/kg on days 1 and 14 of a 28 day cycle) + Glasdegib (100 mg oral daily)	Non-randomized Open-label Phase 1b/2	*n* = 138	December 2024	Naval G. Daver, MD	NCT03390296
R/R AML	Avelumab (starting dose for dose escalation 3.0 mg/kg on days 1 and 14 of 28 day cycle) and Azacytidine (75 mg/m^2^ days 1–7 or days 1–5, 8–9 of 28 day cycle)	Non-randomized Open-label Phase 1b/2	*n* = 58	February 2021	Naval G. Daver, MD	NCT02953561

Legend: R/R = relapsed/refractory; ALL = acute lymphoblastic leukemia; AML = acute myeloid leukemia; MDS = myelodysplastic syndrome; HR = high risk; HSCT = hematopoietic stem cell transplantation.
